# Molecular characterization of the waterborne pathogens *Cryptosporidium* spp., *Giardia duodenalis*, *Enterocytozoon bieneusi*, *Cyclospora cayetanensis* and *Eimeria* spp. in wastewater and sewage in Guangzhou, China

**DOI:** 10.1186/s13071-020-04566-5

**Published:** 2021-01-20

**Authors:** Yingying Fan, Xinrui Wang, Ruohong Yang, Wentao Zhao, Na Li, Yaqiong Guo, Lihua Xiao, Yaoyu Feng

**Affiliations:** 1 Center for Emerging and Zoonotic Diseases, College of Veterinary Medicine, South China Agricultural University, Guangzhou, 510642 Guangdong China; 2 Guangdong Laboratory for Lingnan Modern Agriculture, Guangzhou, 510642 Guangdong China

**Keywords:** *Cryptosporidium*, *Giardia*, *Enterocytozoon bieneusi*, *Eimeria*/*Cyclospora*, Wastewater, Sewer system, WWTP influent, China

## Abstract

**Background:**

The waterborne pathogens *Cryptosporidium* spp., *Giardia duodenalis*, *Enterocytozoon bieneusi* and *Cyclospora cayetanensis* can cause intestinal diseases in humans. An understanding of their occurrence and transport in the environment is essential for accurate quantitative microbial risk assessment.

**Methods:**

A total of 238 influent samples were collected from four wastewater treatment plants (WWTPs) and 88 samples from eight sewer locations in Guangzhou, China. PCR-based tools were used to detect and genetically characterize *Cryptosporidium* spp., *G. duodenalis* and *E. bieneusi*. *Eimeria* spp. and *Cyclospora* spp. were also analyzed to assess the sources of *Cryptosporidium* spp., *G. duodenalis* and *E. bieneusi* in wastewater.

**Results:**

The overall occurrence rates in the WWTP and sewer samples were 14.3% (34/238) and 13.6% (12/88) for *Cryptosporidium* spp., 55.5% (132/238) and 33.0% (29/88) for *G. duodenalis*, 56.3% (134/238) and 26.1% (23/88) for *E. bieneusi* and 45.4% (108/238) and 47.7% (42/88) for *Eimeria* spp., respectively. Altogether, 11 *Cryptosporidium* species and genotypes, six *G. duodenalis* genotypes, 11 *E. bieneusi* genotypes and four *C. cayetanensis* were found, together with the presence of nine *Eimeria* species. The common occurrence of *Cryptosporidium* rat genotype IV, *C. muris* and *Eimeria papillata* and *E. nieschulzi* suggested that rodents were significant sources of the enteric pathogens detected in the wastewater samples.

**Conclusions:**

While the dominant *Cryptosporidium* spp. detected in the raw wastewater sampled in this study are not pathogenic to humans, the widely detected *G. duodenalis* assemblage A and *E. bieneusi* genotypes D and Type IV are well-known zoonotic pathogens. Further studies are needed to monitor the occurrence of these waterborne pathogens in WWTPs to better understand their transmission and environmental transport in China.
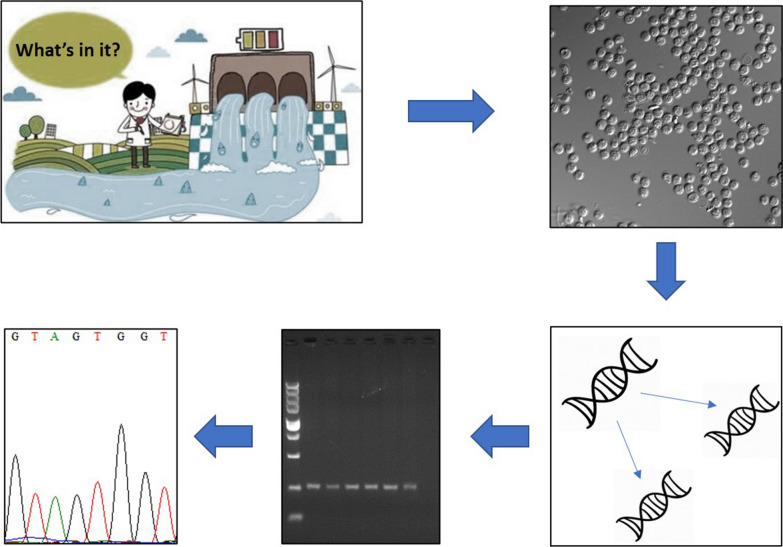

## Background

Waterborne parasitic diseases affect humans in both developed and developing countries [1]. Among these, *Cryptosporidium* spp. and *Giardia duodenalis* are major parasitic protozoa associated with waterborne outbreaks of illnesses in the USA and Europe [2, 3], and many disease outbreaks caused by these pathogens have been reported [4]. For example, worldwide there were 239 reported outbreaks of cryptosporidiosis with approximately 65,540 cases documented between 2011 and 2016 [1], and 280 million cases of giardiasis were reported annually, mostly in Asia, Africa and South America [5]. *Enterocytozoon bieneusi* is a microsporidia commonly found in humans and animals that causes diarrhea in both immunocompetent and immunocompromised individuals [6]. It has been implicated in causing outbreaks of diarrhea in humans [7, 8]. *Cyclospora cayetanensis* is recognized as an emerging food- and waterborne parasite, transmitted by environmentally robust oocysts [9]. These pathogens are unique due to their low infective dose and robust infective stages that are also resistant to many common disinfectants, such as chlorine and chloramines [10, 11]. One illustration of the impact of waterborne parasitic diseases is the massive outbreak of cryptosporidiosis in Milwaukee, Wisconsin in 1993, which caused illness in > 400,000 people and > 100 deaths [12].

Guangzhou, with a population of > 15 million people, is one of the largest cities in China. The subtropical climate and abundant rainfall provide a favorable environment for the transmission of waterborne pathogens. However, a lack of surveillance systems in Guangzhou makes it difficult to investigate the transmission of these pathogens in the major urban environment. Currently, no relevant data are available on the prevalence of enteric parasites in humans and wastewater in the city.

PCR and sequence analyses of several genetic markers, such as the small subunit (*SSU*) of rRNA and 60-kDa glycoprotein (*gp60*) genes for *Cryptosporidium* spp., the internal transcribed spacer (ITS) of the rRNA gene for *E. bieneusi* and the triose phosphate isomerase (*tpi*), glutamate dehydrogenase (*gdh*) and β-giardin (*bg*) genes for *G. duodenalis*, have been used in the detection and characterization of pathogens [3, 13]. They are also useful for the assessment of infection sources and transmission dynamics of cryptosporidiosis, giardiasis and microsporidiosis [3]. Based on data accumulated to date, *Cryptosporidium hominis*, *C. parvum*, *C. meleagridis*, *C. felis*, *C. canis*, *C. muris*, *C. ubiquitum*, *C. cuniculus*, *G. duodenalis* assemblages A and B as well as *E. bieneusi* genotypes A, B, D and Type IV appear to be responsible for the majority of human cases of these diseases worldwide [14–18]. Some of them, such as *C. hominis* and *E. bieneusi* genotypes A and B, are transmitted mostly anthroponotically [19], whereas *C. parvum*, *C. felis*, *C. canis*, *G. duodenalis* and *E. bieneusi* genotypes D and Type IV may be acquired through either through anthroponotic or zoonotic transmission [16, 20]. Therefore, the characterization of pathogen species and genotypes in wastewater could provide insight into the potential infection sources and transmission dynamics of intestinal diseases in urban areas [21].

In the present study, *Cryptosporidium* spp., *G. duodenalis*, *E. bieneusi*, and *Eimeria* spp. present in raw wastewater samples from Guangzhou were characterized at the species and genotype levels to assess the contribution of humans and domestic animals to their occurrence in wastewater. *Eimeria* spp., which commonly infect domestic mammals and birds and have a strong host specificity, were also analyzed to facilitate determination of the contribution of animal sources of *Cryptosporidium* spp., *G. duodenalis*, and *E. bieneusi* found in wastewater [21].

## Methods

### Specimens

A total of 88 grab samples (500–1000 ml per sample) of raw wastewater were collected at weekly intervals between August 2018 and October 2018 from eight sites of the sewer distribution system located in Guangzhou, China. In addition, 238 raw wastewater samples were collected weekly between November 2018 and February 2019 from four wastewater treatment plants (WWTPs) in the city, including 66 from WWTP1, 28 from WWTP2, 66 from WWTP3 and 78 from WWTP4. All WWTPs examined in the study were located in areas of high population density where a high occurrence of rats and other rodents in the wastewater distribution system had been noted. All wastewater samples were collected into 1000-ml plastic bottles, stored on ice and transported to the laboratory immediately where the pathogens in the samples were concentrated by centrifugation at 1500*g* for 20 min, following which the sediment was collected and stored at 4 °C in 2.5% potassium dichromate solution prior to DNA isolation.

### DNA extraction and pathogen detection by PCR

Following washing of the sample concentrates twice with distilled water to remove the potassium dichromate by centrifugation, genomic DNA was extracted from 0.5 ml of the concentrate using the FastDNA Spin Kit for Soil (MP Biomedical, Santa Ana, CA, USA) and then eluted with 100 μl reagent-grade water. Each sample was analyzed at least three times by nested PCR at each genetic locus, using 2 μl of the genomic DNA as the template for amplification. Positive, negative and no template controls were included in each PCR run. The secondary PCR products were analyzed by 1.5% agarose electrophoresis.

### Detection, genotyping and subtyping of *Cryptosporidium* spp.

A nested PCR targeting an ~ 830-bp fragment of the *SSU* rRNA gene was used to detect *Cryptosporidium* spp. [22]. The *Cryptosporidium* spp. present in the samples were identified to the species level by sequence analysis of the secondary PCR products. The *C. hominis*, *C. parvum* and *C. meleagridis* thus identified were subtyped by PCR and sequence analysis of an ~ 850-bp fragment of the *gp60* gene as described previously [23, 24].

### Detection, genotyping and subtyping of *G. duodenalis*

The presence of* G. duodenalis* was determined by running three nested PCR assays targeting a 530-bp fragment of the *tpi* gene [25], a 599-bp fragment of the *gdh* gene [26] and a 511-bp fragment of the *bg* gene [27]. Assemblages and sub-assemblages of *G. duodenalis* were determined using sequence analysis of the secondary PCR products.

### Detection and genotyping of *E. bieneusi*

A nested PCR analysis of a 392-bp fragment of the rRNA gene containing the entire ITS was used to detect *E. bieneusi* [28]. Genotypes of *E. bieneusi* were determined by sequence analysis of the secondary PCR products. The established nomenclature system was used in naming *E. bieneusi* genotypes [29].

### Detection and identification of *Eimeria* spp. and *Cyclospora* spp.

*Eimeria* spp. and *Cyclospora* spp. were detected in a nested PCR analysis which amplified a 294-bp fragment of the *SSU* rRNA gene, as well as DNA of the genetically related *Isospora* spp. [30]. The identification of *Eimeria* and *Cyclospora* species was done by sequence analysis of the secondary PCR products.

### DNA sequence analysis

All positive secondary PCR products generated from the study were sequenced in both directions on an ABI 3170 Genetic Analyzer (Applied Biosystems, Foster City, CA, USA). The sequences were assembled using ChromasPro 1.33 (www.technelysium.com.au/ChromasPro.html), edited using BioEdit 7.04 (www.mbio.ncsu.edu/BioEdit/bioedit.html) and aligned using ClustalX 2.1 (www.clustal.org/). The concurrent presence of mixed species or genotypes of the pathogens under analysis was determined by discordant results among the three PCR replicates. To assess the phylogenetic placement of *E. bieneusi* genotypes found in this study, we constructed a maximum-likelihood tree using MEGA 6.0 (www.megasoftware.net/). The general time-reversible model and gamma distribution were used in the calculation of substitution rates, and 1000 replicates were used in bootstrap analysis of the phylogenetic tree.

### Statistical analysis

The frequency of pathogen occurrence between sampling locations was compared using the χ^2^ test implemented in SPSS 19.0 for Windows (IBM Corp., Armonk, NY, USA). Differences with *P* values of < 0.05 were considered to be significant.

## Results

### Occurrence of *Cryptosporidium* spp. in wastewater

The PCR analysis revealed that 34 (14.3%) wastewater samples from WWTPs and 12 (13.7%) sewer samples from the sewer distribution system were positive for *Cryptosporidium* spp. The difference in *Cryptosporidium* occurrence between the samples from the WWTPs and sewer system was not significant (χ^2^ = 0.022, *df* = 1, *P* = 0.8821). Among the samples collected from the four WWTPs, *Cryptosporidium* spp. were detected in 19.7% of samples from WWTP1, 21.4% from WWTP2, 27.3% from WWTP3 and 11.5% from WWTP4 (χ^2^ = 5.81, *df* = 1, *P* = 0.0159; Table 1). Among samples from the eight sewer sampling sites, the occurrence rates of *Cryptosporidium* spp. varied from 0 and 27.3%. The difference in *Cryptosporidium* occurrence among the eight sewer sampling sites was not significant (χ^2^ = 1.54, *df* = 1, *P* = 0.0623).

**Table 1:** Occurrence and distribution of *Cryptosporidium* species in samples collected from wastewater treatment plants and in sewer samples, Guangzhou, China

Sample location	No. of samples	No. of positive samples (%)	*Cryptosporidium* species (no. of samples)
WWTP1	66	13 (19.7%)	*C. baileyi* (3); rat genotype IV (3); *C. meleagridis* (2); *C. muris* (2); *C. bovis* (1); *C. occultus* (1); *C. serpentis* (1)
WWTP2	28	6 (21.4%)	*C. muris* (3); *C. occultus* (1); *C. canis* (1); *C. parvum* (1)
WWTP3	66	18 (27.3%)	*C. baileyi* (5); *C. bovis* (3); *C. muris* (3); *C. felis* (2); rat genotype IV (2); *C. occultus* (1); *C. parvum* (1); rat genotype I (1)
WWTP4	78	9 (11.5%)	Rat genotype IV (5); *C. muris* (4)
Sub-total	238	46 (14.3%)	*C. muris* (12); rat genotype IV (10); *C. baileyi* (8); *C. bovis* (4); *C. occultus* (3); *C. meleagridis* (2); *C. parvum* (2); *C. felis* (2); *C. serpentis* (1); *C. canis* (1); rat genotype I (1)
Sewer 1	11	1 (9.1)	Rat genotype IV (1)
Sewer 2	11	0	–
Sewer 3	11	0	–
Sewer 4	11	1 (9.1)	Rat genotype IV (1)
Sewer 5	11	1 (9.1)	Rat genotype IV (1)
Sewer 6	11	3 (27.7)	*C. felis* (3)
Sewer 7	11	3 (27.3)	*C. felis* (2), *C. parvum* (1)
Sewer 8	11	3 (27.3)	Rat genotype IV (1), *C. parvum* (1), *C. baileyi* (1)
Sub-total	88	12 (13.6%)	*C. felis* (5); Rat genotype IV (4); *C. parvum* (2); *C. baileyi* (1)
Total	326	58 (17.8%)	Rat genotype IV (14); *C. muris* (12); *C. baileyi* (9); *C. felis* (7); *C. parvum* (4); *C. bovis* (4); *C. occultus* (3); *C. meleagridis* (2); *C. serpentis* (1); *C. canis* (1); rat genotype I (1)

WWTP, Wastewater treatment plant

Sequence analysis of the PCR products revealed the presence of 11 *Cryptosporidium* species and genotypes in samples collected from the WWTPs, including *C. muris* (*n* = 12), rat genotype IV (*n* = 10), *C. baileyi* (*n* = 8), *C. bovis* (*n* = 4), *C. occultus* (*n* = 3), *C. meleagridis* (*n* = 2), *C. parvum* (*n* = 2), *C. felis* (*n* = 2), *C. serpentis* (*n* = 1), *C. canis* (*n* = 1) and rat genotype I (*n* = 1). No concurrent presence of multiple *Cryptosporidium* species or genotypes was detected. At least one *Cryptosporidium* species was detected in each WWTP. The two *C. parvum*-positive samples from WWTP2 and WWTP3 were identified being subtype IIdA15G1.

Four *Cryptosporidium* species were identified in samples from the sewer system, including *C. felis* (*n* = 5), rat genotype IV (*n* = 4), *C. parvum* (*n* = 2) and *C. baileyi* (*n* = 1). For *C. parvum*, only one of the two positive samples from sewer site 8 was successfully subtyped, yielding IIdA15G1. No concurrent presence of multiple *Cryptosporidium* species was detected.

### Occurrence of *G. duodenalis* assemblages and sub-assemblages in wastewater

Among the 238 wastewater samples collected from the WWTPs, 55.5% (132/238), 52.1% (124/238) and 53.4% (127/238) were PCR positive for *G. duodenalis* at the *gdh*, *tpi* and *bg* loci, respectively. Similarly, among the 88 samples collected from the sewer system, 31.8% (28/88), 25.0% (22/88) and 25.0% (22/88) were PCR positive at these loci, respectively. The difference in *G. duodenalis* occurrence between samples from the WWTPs and sewer system was significant (χ^2^ = 14.371, *df* = 1, *P* = 0.0002). The most commonly detected genotype in the WWTP samples and the sewer system samples was assemblage A (*n* = 124 and 19 respectively). As subtype A2 was identified at all three genetic loci, the assemblage A belonged to the sub-assemblage AII. In addition, assemblages B (*n* = 4), G (*n* = 3) and C (*n* = 1) were seen in a few WWTP samples. Similarly, assemblages B (*n* = 5), F (*n* = 2) and D (*n* = 1) were detected in some sewer samples. The presence of multiple *G. duodenalis* assemblages were identified in four samples by identifying different assemblages in replicate PCR analyses of DNA (2 assemblages with A2 + B, 1 with A2 + D and 1 with A2 + F) (Table 2).

**Table 2:** *Giardia duodenalis* assemblages in samples collected from wastewater treatment plants and in sewer samples, Guangzhou, China

Sample location	No. of samples	No. of positive samples (%)			Assemblage (no. of samples)		
		*gdh*	*tpi*	*bg*	*gdh*	*tpi*	*bg*
WWTP1	66	42	37	37	A (37); G (3); B (2)	A (34); B (2); A + B (1)	A (33); B (3); A + B (1)
WWTP2	28	19	19	19	A (19)	A (19)	A (19)
WWTP3	66	29	29	32	A (27); C (1); B (1)	A (28); C (1)	A (31); C (1)
WWTP4	78	42	39	39	A (41); B (1)	A (38); B (1)	A (38); B (1)
Sub-total	238	132 (55.5%)	124 (52.1%)	127 (53.4%)	A (124); B (4); G (3); C (1)	A (117); B (3); C (2); A + B (1)	A (117); B (3); C (1); A + B (1)
Sewer 1	11	5	5	5	A (4); B (1)	A (5)	A (5)
Sewer 2	11	0	0	0			
Sewer 3	11	0	0	0			
Sewer 4	11	0	0	0			
Sewer 5	11	1	0	1	D (1)		D (1)
Sewer 6	11	6	5	4	A (6)	A (3); B (1); F (1)	A (4)
Sewer 7	11	6	5	5	A (3); B (2); A + B (1)	A (2); B (3)	A (3); B (2)
Sewer 8	11	10	7	7	A (6); B (1); A + B (1); A + D (1); A + F (1)	A + B (1); A (5); B (1); A + B (1)	A (5); B (1); D (1)
Sub-total	88	28 (31.8%)	22 (25.0%)	22 (25.0%)	A (19); B (4); A + B (2); D (1); A + D (1); A + F (1)	A (15); B (5); F (2), A + B (1)	A (17); B (3); D (2)
Total	326	160 (49.1%)	146 (44.8%)	149 (45.7%)	A (143); B (8); G (3); C (1); D (1); A + B (2); A + D (1); A + F (1)	A (132); B (8); C (2); F (2), A + B (2)	A (134); B (6); C (1); D (2), A + B (1)

* bg* β-Giardin gene;* gdh*, glutamate dehydrogenase gene;* gp60*, 60-kDa glycoprotein gene

### Occurrence of *Enterocytozoon bieneusi* genotypes in wastewater

PCR analysis of the ITS locus revealed the presence of *E. bieneusi* in 56.3% (34/238) of WWTP samples and 26.1% (23/88) of sewer samples (Table 3). The difference between the two groups was significant (χ^2^ = 23.417, *df *= 1, *P  *< 0.0001). Among the samples from the four WWTPs, the highest occurrence was in those collected from WWTP2 (67.9%), followed by WWTP3 (57.6%), WWTP4 (53.9%) and WWTP1 (53.0%). The difference between WWTPs, however, was not significant (χ^2^ = 1.768, *df *= 1, *P* = 0.184). Among the eight sewer sampling sites, sites 7 and 8 showed the highest occurrence of *E. bieneusi* (both 63.6%).

**Table 3:** Internal transcribed spacer genotypes of *Enterocytozoon bieneusi* in samples collected from wastewater treatment plants and in sewer samples, Guangzhou, China

Sample location	No. of samples	No. of positive samples (%)	Genotypes (no. of samples)
WWTP1	66	35 (53.0%)	D (27); Peru8 (4); Type IV + D (2); GZW1^a^ (1); EbpC (1)
WWTP2	28	19 (67.9%)	D (11); Type IV (5); Type IV + D (2); Peru8 + Type IV (1)
WWTP3	66	38 (57.6%)	D (22); Type IV (11); Type IV + D (1); Peru8 (2); Peru6 (1); PtEb IX (1)
WWTP4	78	42 (53.9%)	D (17); Type IV (14); Peru8 (4); Peru11 (2); Type IV + D (2); EbpC (1); Type IV + Peru11 (1); MWC-m1 (1)
Sub-total	238	134 (56.3%)	D (77); Type IV (30); Peru8 (10); Peru11 (2); EbpC (2); Peru6 (1); MWC-m1 (1); GZW1^a^ (1); PtEb IX (1); Type IV + D (7); Type IV + Peru11 (1); Peru8 + Type IV (1)
Sewer 1	11	2 (18.2%)	D (2)
Sewer 2	11	1 (9.1%)	D (1)
Sewer 3	11	0	–
Sewer 4	11	1 (9.1%)	GZW2^a^ (1)
Sewer 5	11	1 (9.1%)	D (1)
Sewer 6	11	4 (36.4%)	D (3); Type IV (1)
Sewer 7	11	7 (63.6%)	D (3); Type IV (1); PtEb IX (2), GZW3a (1)
Sewer 8	11	7 (63.6%)	Type IV (3); D (1); Type IV + D (2); EbpC (1)
Sub-total	88	23 (26.1%)	D (11); Type IV (5); PtEb IX (2); Type IV + D (2); EbpC (1); GZW2^a^ (1); GZW3^a^ (1)
Total	326	157 (48.2%)	D (88); Type IV (35); Peru8 (10); Type IV + D (9); PtEb IX (3); EbpC (3); Peru11 (2); Type IV + Peru11 (1); Peru6 (1); MWC-m1 (1); GZW1^a^ (1); GZW2^a^ (1); GZW3^a^ (1); Peru8 + Type IV (1)

^a^Novel genotype found in this study

Among the 134 *E. bieneusi*-positive samples from the WWTPs, nine genotypes of *E. bieneusi* were identified, including D (*n* = 77), Type IV (*n* = 30), Peru8 (*n* = 10), Peru11 (*n* = 2), EbpC (*n* = 2), Peru6 (*n* = 1), MWC-m1 (*n* = 1) and PtEb IX (*n* = 1). A novel genotype was detected and named GZW1 (*n* = 1). In addition, nine samples were found to have concurrent presence of two genotypes, including Type IV + Peru11 in seven samples, Peru8 + Type IV in one sample and Type IV + D in one sample (Table 3).

Among 23 *E. bieneusi*-positive sewer samples, six genotypes were detected, including D (*n* = 11), Type IV (*n* = 5), PtEb IX (*n* = 2), EbpC (*n* = 1) and two novel genotypes named GZW2 (*n* = 1) and GZW3 (n = 1). In addition, the concurrent presence of two genotypes (Type IV + D) was detected in two sewer samples.

**Fig. 1:**
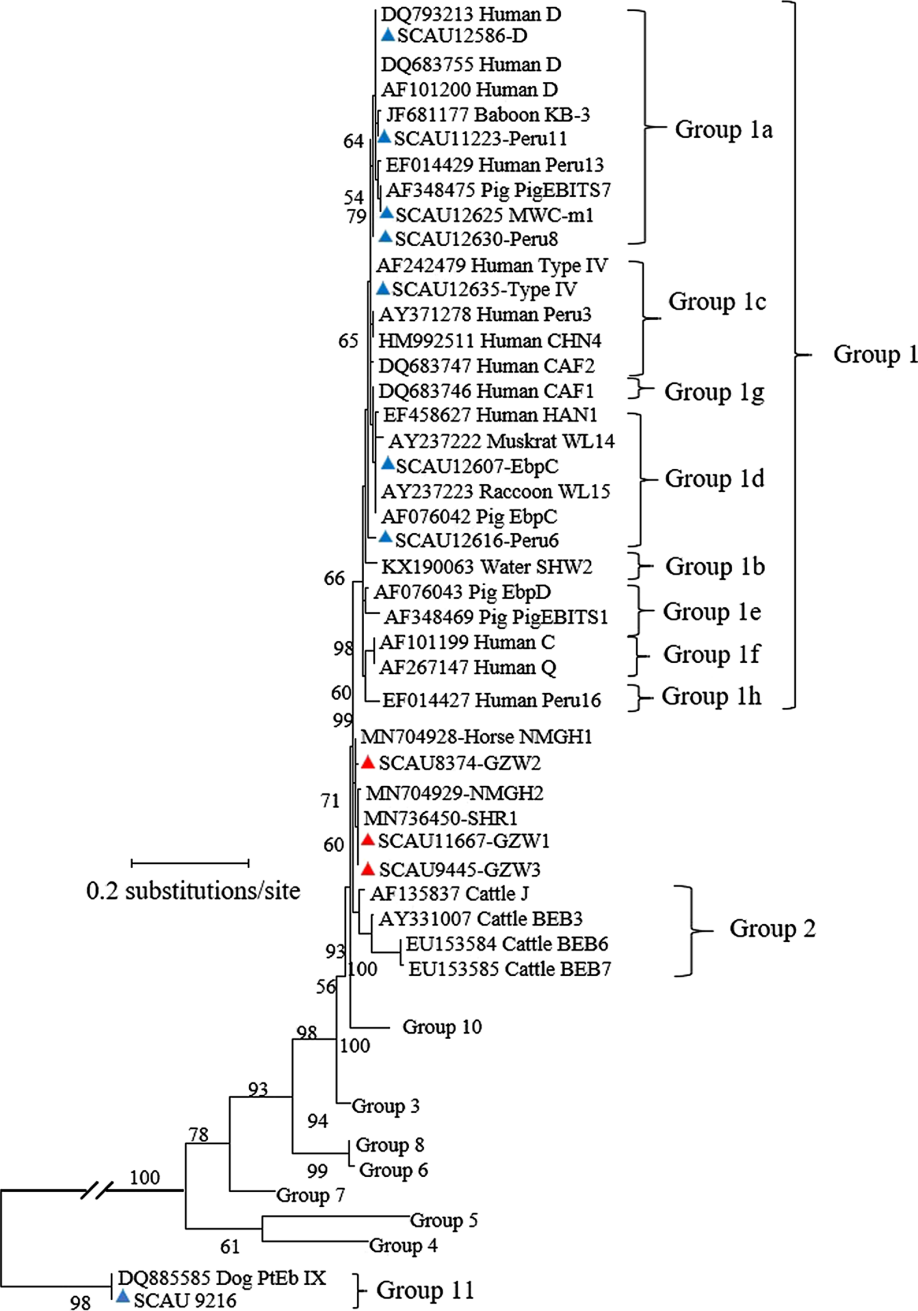
Phylogenetic relationship of *Enterocytozoon bieneusi* genotypes detected in samples collected from wasterwater treatment plants and sewer samples as determined by a maximum-likelihood analysis of the ribosomal internal transcribed spacer based on substitution rates calculated using the general time-reversible model and gamma distribution. Known and novel genotypes identified in this study are indicated by blue and red triangles, respectively. Bootstrap values of < 50% from 1000 replicate analysis are not shown

In the maximum likelihood analysis of the ITS sequences obtained, genotypes D Type IV, Peru11, EbpC, Peru6 and Peru8 were clustered within Group 1, and PtEb IX within Group 11, while the three novel genotypes GZW1, GZW2 and GZW3 formed a new clade between Groups 1 and 2 (Fig. 1).

### Occurrence of *Eimeria* spp. and *Cyclospora* spp. in wastewater

For *Eimeria* spp. or *Cyclospora* spp., the occurrence rates were 45.4% (108/238) and 47.7% (42/88) in WWTP and sewer samples, respectively. The difference in occurrence rates between these was not significant (Table 4; χ^2^ = 0.143, *df* = 1, *P* = 0.7053). The majority of the PCR products (141/150, 94.0%) in the WWTP samples were from *Eimeria* spp., including *E. papillata* (*n* = 54), *E. nieschulzi* (*n* = 30), *E. necatrix* (*n* = 6), *E. falciformis* (*n* = 6), *E. polita* (*n* = 1), *E. mitis* (*n* = 1), *E. acermlina* (*n* = 1) and *E. polita* (*n* = 1). In addition, five samples showed concurrent presence of two *Eimeria* species; these *Eimeria* species were also found in sewer samples (Table 4). *Cyclospora cayetanensis*, however, was detected in three sewer samples and one WWTP sample (4/150; 2.7%). In addition, *Isospora* spp. were found using the PCR in three sewer samples and two WWTPs (Table 4).

**Table 4:** *Eimeria* species and related parasites in samples collected from wastewater treatment plants and in sewer samples, Guangzhou, China

Sample location	No. of samples	No. of positive samples	Species (no. of samples)
WWTP1	66	36 (54.6%)	*E. papillata* (21); *E. nieschulzi* (13); *E. falciformis* (2)
WWTP2	28	7 (50.0%)	*E. papillata* (4); *E. polita* (1); *E. nieschulzi* (1); *Isospora* sp. (1)
WWTP3	66	26 (39.4%)	*E. papillata* (13); *E. nieschulzi* (5); *E. falciformis* (2); *E. necatrix* (1); *E. mitis* (1); *E. acermlina* (1); *E. nieschulz* + *E. falciformis* (1); *E. nieschulz* + *E. papillata* (1); *C. cayetanensis* (1)
WWTP4	78	39 (50.0%)	*E. papillata* (16); *E. nieschulzi* (11); *E. necatrix* (5); *E. papillata* + *E. necatrix* (3); E*. falciformis* (2); *E. polita* (1); *Isospora* sp. (1)
Sub-total	238	108 (45.4%)	*E. papillata* (54); *E. nieschulzi* (30); *E. necatrix* (6); *E. falciformis* (6); *E. papillata* + *E. necatrix* (4); *E. nieschulz* + *E. falciformis* (1); *Isospora* sp. (2); *E. polita* (1); *E. mitis* (1); *E. acervulina* (1); *E. polita* (1); *C. cayetanensis* (1)
Sewer 1	11	6 (54.6%)	*E. nieschulzi* (4); *E. papillata* (1); *Isospora* sp. (1)
Sewer 2	11	2 (18.2%)	*E. nieschulzi* (1); *E. falciformis* (1);
Sewer 3	11	1 (9.1%)	*E. nieschulzi* (1)
Sewer 4	11	9 (81.8%)	*E. nieschulzi* (6); *E. papillata* (1); *Isospora* sp. (1); *E. falciformis* (1)
Sewer 5	11	3 (27.3%)	*E. nieschulzi* (1); *E. papillata* (1); *Isospora* sp. (1)
Sewer 6	11	8 (72.7%)	*C. cayetanensis* (3); *E. nieschulzi* (2); *E. papillata* (2); *E. falciformis* (1)
Sewer 7	11	6 (54.6%)	*E. nieschulzi* (3); *E. papillata* (3)
Sewer 8	11	7 (63.6%)	*E. nieschulzi* (4); *E. papillata* (2)
Sub-total	88	42 (47.7%)	*E. nieschulzi* (22); *E. papillata* (10); *C. cayetanensis* (3); *E. falciformis* (3); *Isospora* sp. (3)
Total	326	150 (46.0%)	*E. papillata* (64); *E. nieschulzi* (52); *E. falciformis* (9); *E. necatrix* (6); *Isospora* sp. (5); *E. polita* (1); *E. mitis* (1); *E. acervulina* (1); *E. polita* (1); *E. papillata* + *E. necatrix* (4); *E. nieschulz* + *E. falciformis* (1); *C. cayetanensis* (4)

## Discussion

The results of the present study provide some preliminary data on the occurrence of zoonotic waterborne pathogens (i.e. *Cryptosporidium* spp., *G. duodenalis* and *E. bieneusi*) in the wastewater of Guangzhou, China. Prior to this study, data on the occurrence of these pathogens in WWTP samples were available from Harbin, Qingdao, Nanjing, Shanghai and Wuhan [21, 31–33]. In this study, we detected *Cryptosporidium* spp., *G. duodenalis*, *E. bieneusi* and *Eimeria* spp. in 14.1, 49.1, 49.7 and 43.3%, respectively, of all wastewater samples collected. Overall, the occurrence rates of these pathogens were lower than those reported from the studies previously conducted in China [21, 31]. The reasons for this difference are as yet clear, but differences in the number of samples analyzed, geography and habitat, wastewater treatment practices and health status of local residents may be contributing factors.

As seen in this study, 11 *Cryptosporidium* species and genotypes were detected in samples collected from WWTPs. Among these, *C. muris* (which infects a range of rodents, other mammals and humans) was the most common species (in 33% or 11 of the 34 *Cryptosporidium*-positive samples). This result is in agreement with the frequent detection of rat genotype IV (10 of 34 *Cryptosporidium*-positive samples), suggesting that rodents contribute significantly to the occurrence of *Cryptosporidium* spp. in wastewater systems in Guangzhou. In previous studies in China [32], Greece [34] and Brazil [35], rodents were identified as a major source of *Cryptosporidium* oocysts in wastewater. Nevertheless, the predominance of *C. muris* and rat genotype IV in the present study contrasts with the dominance of *C. hominis* in wastewater reported in previous studies conducted in China [21, 31, 36]. This difference may either be due to differences in the sampling scheme or differences in the transmission of *Cryptosporidium* spp. among areas in China. It should be mentioned that there was no concurrent presence of multiple *Cryptosporidium* species/genotypes detected in the present study, possibly due to the relatively lower occurrence rates of these pathogens. The direct Sanger sequencing used in the analysis of PCR products, however, is not ideal for the detection of mixed *Cryptosporidium* spp. in samples. Next-generation sequencing techniques have been demonstrated to have better capability of detecting mixed populations of *Cryptosporidium* spp. in water samples [37].

*Cryptosporidium muris* was identified in the present study. Although it is not a major zoonotic pathogen, the species has gained increased attention in recent years. Several recent studies have documented the occurrence of *C. muris* in humans in developing countries [38–40]. In addition, macaque monkeys in China are commonly infected with *C. muris* [41]. Experimental infection with *C. muris* can cause persistent diarrhea in humans [42]. These findings on *C. muris* and the occurrence of other well-known human pathogen species, such as *C. parvum*, *C. meleagridis*, *C. canis* and *C. felis*, indicate that *Cryptosporidium* spp. wastewater constitute a possible public health issue.

The results of this study show that assemblage A was the dominant *G. duodenalis* genotype in the wastewater samples. This result is consistent with observations in previous studies in Shanghai and elsewhere in China [21, 33, 43], USA, Tunisia and Hungary [44–46], which all reported that this assemblage was the dominant *G. duodenalis* assemblage in wastewater. In addition, all PCR products from the assemblage A-positive samples yielded sequences of the sub-assemblage AII, which is known to be mainly a human pathogen [2]. Other *G. duodenalis* genotypes associated with animals were found in a small number of samples, including assemblages C, D, F and G; this is in agreement with the detection of *C. felis*, *C. canis* and *C. muris* in these wastewater samples.

A high genetic diversity of *E. bieneusi* was detected in the WWTP and sewer samples. Among the 11 known genotypes of *E. bieneusi* detected in this study, the majority was determined to belong to Group 1 (Fig. 1), which contains most *E. bieneusi* genotypes found in humans [16]. The dominant genotype D in the WWTP and sewer samples in this study was also reported to be the dominant *E. bieneusi* genotype in wastewater samples from Shanghai [21, 32, 43], Nanjing, Qingdao and Wuhan [21]. The known hosts for genotype D include humans, cattle, pigs, dogs, goats, sheep, wild mammals and birds [16, 47]. In the present study, the contribution of these animals to genotype D was probably limited, as some of the common host-adapted *E. bieneusi* genotypes of cattle (BEB6 of Group 2), pigs (EbpD of Group 1e) and wild mammals (Groups 3–8) were not identified (Table 3; Fig. 1). As this genotype is especially common in rodents [16, 48–50], rats and mice in the sewer distribution system could be the major source. This possibility is corroborated by the frequent detection of *C. muris*. Several other *E. bieneusi* genotypes identified in the study, such as Type IV and Peru8, are also known to infect rodents [16, 50]. Nevertheless, humans cannot be excluded as a source of contamination with these genotypes [51, 52]. As these *E. bieneusi* genotypes are major zoonotic genotypes, the presence of *E. bieneusi* in wastewater represents a potentially high public health hazard.

The results of the genetic characterization of *Eimeria* spp. support the contribution of rodents to the occurrence of enteric pathogens in wastewater [21]. *Eimeria* spp. are host specific and form host-associated clusters in the phylogenetic analysis of the *SSU* rRNA sequences, which make it possible to track the source of pathogens in environmental samples [21]. In the present study, several rodent *Eimeria* spp., such as *E. papillata* (*n* = 64) and *E. nieschulzi* (*n* = 51), were commonly detected in wastewater samples, corroborating the contribution of rodents to the occurrence of *Cryptosporidium* spp. and *E. bieneusi*.

Although the detection of *C. cayetanensis* in wastewater samples in the present study was sporadic, the presence of this species represents a significant finding. *Cyclospora cayetanensis* is an emerging parasitic pathogen responsible for numerous foodborne outbreaks of cyclosporiasis in humans in industrialized countries [53]. Thus far, it has only been occasionally reported in humans in Anhui and Henan, China [54, 55]. The finding of *C. cayetanensis* in some samples in the study reinforces the contribution of humans to the occurrence of enteric parasites in wastewater.

## Conclusions

The results of the present study provide some insight into the genetic make-up of *Cryptosporidium* spp., *Giardia duodenalis*, *Enterocytozoon bieneusi*, *Eimeria* spp. and *Cyclospora* spp. in the wastewater of Guangzhou, China. With the exception of *G. duodenalis*, *E. bieneusi* genotypes Type IV and D, and *C. cayetanensis*, some of the pathogens detected do not appear to originate from humans. Rodents in particular probably contribute significantly to the occurrence of these enteric pathogens in wastewater. Given the public health risk to humans that these parasites represent, guidelines on the discharge and reuse of wastewater are needed to reduce the transmission and environmental transport of these pathogens in areas with limited water sources. Future, more large-scale and long-term surveys of wastewater are needed to generate key information needed for an improved understanding of the transmission and environmental transport of enteric pathogens in urban communities.

## Data Availability

Data supporting the conclusions of this article are included within the article. Representative nucleotide sequences generated in this study were deposited in the GenBank database under the accession numbers MW082597-MW082599, MW089000-MW089010 and MW090927-MW090943.

## Abbreviations

*bg*: β-Giardin gene
*gdh*: Glutamate dehydrogenase gene
*gp60*: 60-kDa glycoprotein gene
ITS: Internal transcribed spacer
PCR: Polymerase chain reaction
*SSU* rRNA: Small subunit ribosomal RNA gene
*tpi*: Triosephosphate isomerase
